# Semicontinuous Microemulsion Polymerization of Polymeric Nanoparticles of Poly(cyanoacrylates) and Poly(caprolactone)

**DOI:** 10.3390/molecules30132668

**Published:** 2025-06-20

**Authors:** Gerardo León-Sánchez, Eulogio Orozco-Guareño, Oscar Guillermo Zúñiga-González, Luisa Fernanda Briones-Márquez, Raúl R. Quiñonez-López, Jesús Baudelio Campos-García, María de Jesús Palacios-Sánchez

**Affiliations:** 1 Departamento de Procesos Tecnológicos e Industriales, Instituto Tecnológico de Estudios Superiores de Occidente, Periférico Sur Manuel Gómez Morín # 8585, Guadalajara 45604, Jalisco, Mexico; gleon@iteso.mx; 2 Laboratorio de Fisicoquímica, Departamento de Química, Centro Universitario de Ciencias Exactas e Ingenierías, Universidad de Guadalajara, Blvd. Marcelino García Barragán # 1421, esq. Calzada Olímpica, Guadalajara 44430, Jalisco, Mexico; eulogio.orozco@academicos.udg.mx (E.O.-G.); guillermo.zuniga@academicos.udg.mx (O.G.Z.-G.); fernanda.briones@academicos.udg.mx (L.F.B.-M.); draul.quinonez@academicos.udg.mx (R.R.Q.-L.); 3 Departamento de Química, Universidad Autónoma de Aguascalientes, Av. Universidad # 940, Ciudad Universitaria, Aguascalientes 20100, Aguascalientes, Mexico; jesus.campos@edu.uaa.mx

**Keywords:** polymeric nanoparticles, poly(cyanoacrylates), poly(caprolactone), semicontinuous microemulsion polymerization

## Abstract

Polymeric nanoparticles based on poly(ethyl cyanoacrylate) (PECA) and poly(ε-caprolactone) (PCL) were synthesized via semicontinuous microemulsion polymerization for potential biomedical applications. A systematic evaluation of four surfactants (Tween 80, Alkonat L70, Genapol LRO, and Brij-20) was carried out to determine their effects on micelle formation and particle size. Brij-20 enabled the formation of nanoparticles under 100 nm, with optimal conditions identified at 4% surfactant concentration and pH 1.75. The polymerization process included acid-catalyzed ring-opening of ε-caprolactone, followed by the semicontinuous addition of ethyl-2-cyanoacrylate under an inert atmosphere. Copolymerization was confirmed through FT-IR spectroscopy, nuclear magnetic resonance (NMR) spectroscopy, and differential scanning calorimetry, revealing a glass transition temperature (*T*_g_) of 110.9 °C, indicating PECA as the dominant phase. Thermogravimetric analysis showed two decomposition events corresponding to each polymer. Transmission electron microscope analysis revealed nanoparticles averaging 51.74 nm in diameter. These findings demonstrate the feasibility of producing PECA-PCL nanoparticles with controlled size and composition, suitable for drug delivery and other biomedical uses.

## 1. Introduction

Over the past few decades, research on controlled drug delivery systems has intensified to enhance therapeutic efficacy while minimizing side effects [1,2]. Among these systems, colloidal carriers such as liposomes and nanoparticles have shown great potential in modifying drug pharmacokinetics and improving bioavailability [3,4,5]. Polymeric nanoparticles, in particular, offer advantages in terms of stability and controlled release, especially when synthesized via microemulsion polymerization [6]. The use of nanoparticles in blood must be approached with extreme caution, since it has been reported that there are important factors that control the pharmacokinetics of the application of nanoparticles intravenously, since nanoparticles are coated with blood plasma proteins [7]. Also, studies on the cytotoxicity of some polymers in vitro have been carried out [8]. The use of a novel copolymerization of ethyl-2-cyanoacrylate and ε-caprolactone, targeting biomedical applications such as drug encapsulation and controlled release, but with an appropriate methodology to avoid complications in its application [7,8,9]. Poly(ε-caprolactone) (PCL) is a semicrystalline, hydrophobic polymer with a relatively polar ester group and five non-polar methylene groups, making it attractive for drug delivery applications [10]. However, its slow degradation rate and limited biocompatibility may restrict its clinical use. In contrast, poly(alkyl cyanoacrylates) (PECAs) have demonstrated a strong capacity to entrap a wide range of bioactive compounds and release them through biodegradation, though they suffer from poor stability and rapid hydrolysis under aqueous or high-temperature conditions [11]. By copolymerizing these two monomers, this work seeks to combine their favorable properties, enhance nanoparticle stability, and optimize the degradation profile for improved pharmaceutical performance [12].

## 2. Results and Discussion

### 2.1. Analysis of (PECA-PCL) Nanoparticles

#### 2.1.1. Micelle Size Analysis

Based on the analysis performed using QLS in Table 1, the micelle size resulting from the synthesis of nanoparticles (PECA-PCL) with different monomer and surfactant ratios was evaluated. A one-way ANOVA statistical analysis was conducted using the obtained data to determine whether there was a significant difference among the surfactants used. The detailed results are presented in Tables S1–S3 of the Supplementary Material. The ANOVA table breaks down the variance in micelle size into two components: between-group variance and within-group variance. The F-ratio, which in this case is 48.542, represents the ratio of the between-group estimate to the within-group estimate. Since the *p*-value of the F-test is less than 0.05, there is a statistically significant difference in the average micelle size between at least two surfactant levels at the 95.0% confidence level. This demonstrates that the micelle size is affected by the type of surfactant used.

**Figure 1:**
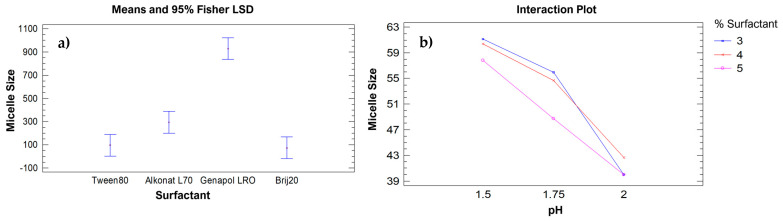
(**a**) Mean plot illustrating the effect of different surfactants on micelle size. (**b**) Interaction plot showing the combined effect of surfactant concentration and pH on micelle size.

The Multiple Range Test shows the presence of three homogeneous groups. The first group consists of the surfactants Tween 80 and Brij 20; the second group includes the surfactant Alkonat L70; and the third group consists of Genapol LRO. This indicates that there is no significant difference in micelle size between Brij 20 and Tween 80. Figure 1 presents the means plot, which shows that the lower limits of the micelle sizes for the first homogeneous group (Tween 80 and Brij 20) are below 100 nm. Based on this result, Brij 20 was selected as the surfactant of choice, along with a 1:1 ratio of the target monomers.

#### 2.1.2. Surfactant Percentage and pH Analysis

Using Brij20 as the surfactant and a 1:1 ratio of ethyl-2-cyanoacrylate and ε-caprolactone, the pH and surfactant concentration were varied to achieve improved results. Table 1 presents the percentage of Brij20 used at different pH values, along with the corresponding average micelle sizes obtained through QLS measurements.

A multivariable ANOVA statistical analysis was performed to determine whether there were significant differences when varying the parameters within the established ranges. The details of the statistical analysis can be found in Tables S4 and S5 of the Supplementary Material. Since Type III sum of squares has been selected (by default), the contribution of each factor is measured after removing the effects of the other factors. The *p*-values test the statistical significance of each individual factor. Since the *p*-value for the pH effect is less than 0.05, this factor has a statistically significant effect on micelle size at a 95.0% confidence level. Multiple range tests were conducted to individually analyze the effect of surfactant percentage and pH. Details of these analyses can be found in Tables S6 and S7 and Figures S1 and S2 in the Supplementary Material. The multiple range test for micelle size by surfactant percentage shows the presence of a single homogeneous group, indicating that there is no significant difference when using 3%, 4%, or 5% surfactant in the synthesis of the copolymer. Similarly, the multiple range test for micelle size by pH effect reveals three different homogeneous groups, corresponding to each of the pH values tested. Figure 1b shows the interaction between surfactant percentage and pH, in relation to micelle size. It can be observed that the smallest micelle size is obtained at pH 2, using either 3% or 5% surfactant in the synthesis.

A fundamental aspect of the analysis through quasi-elastic light scattering is that it allows for the assessment of size uniformity among the micelles in the studied solution, in order to establish the optimal conditions for the synthesis of copolymeric nanoparticles. The graphs obtained based on the variation in the parameters discussed in this section are shown in Figures S3–S11 of the Supplementary Material. From this information, it can be concluded that the lower the pH, the smaller the size distribution among the desired nanoparticles and that the smallest and most uniform nanoparticles were obtained with 4% Brij20 at a pH of 1.75.

#### 2.1.3. Analysis of the PECA-PCL Copolymer via Infrared Spectroscopy (FT-IR)

**Figure 2:**
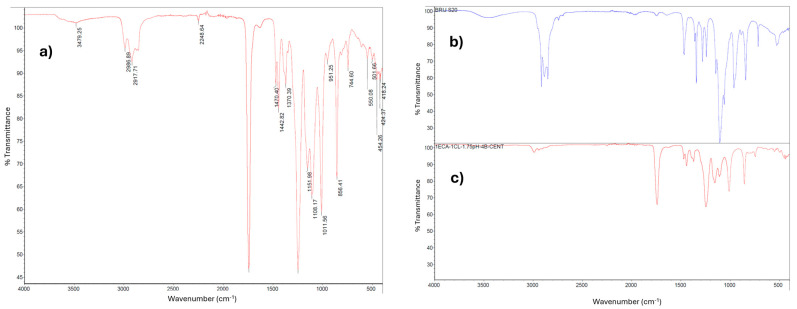
Infrared spectra of (**a**) 1PECA:1PCL, 4Brij20, pH = 1.75; (**b**) pure Brij20; and (**c**) PECA-PCL after centrifugation washes.

The PECA-PCL copolymer was analyzed via infrared spectroscopy to identify the functional groups of the molecule. In Figure 2a, the spectrum of the synthesized PECA-PCL copolymer is shown, where characteristic peaks of the C-N bond at 2248 cm^−1^ from polyethylene cyanoacrylate are observed. Then, around 1741 cm^−1^, a characteristic peak of a carbonyl group attributed to polycaprolactone is seen, confirming that the methodology used is suitable for achieving copolymerization.

At the end of nanoparticle synthesis, a microemulsion in solution was obtained, which had to be broken by removing the surfactant through centrifugation. After the procedure outlined in Section 3.2.2., both the pure Brij20 surfactant (Figure 2b) and the copolymer after washing (Figure 2c) were analyzed via IR spectroscopy to determine if the washes with ethanol and centrifugation were effective. In the spectra in Figure 2b,c, the functional groups previously mentioned in Figure 2a can be observed on a smaller scale, and there is no signal resembling that of the copolymer after washing and the Brij20 surfactant, indicating that the washes were effective.

#### 2.1.4. ^1^H NMR and ^13^C NMR Analysis of the PECA-PCL Copolymer

**Figure 3:**
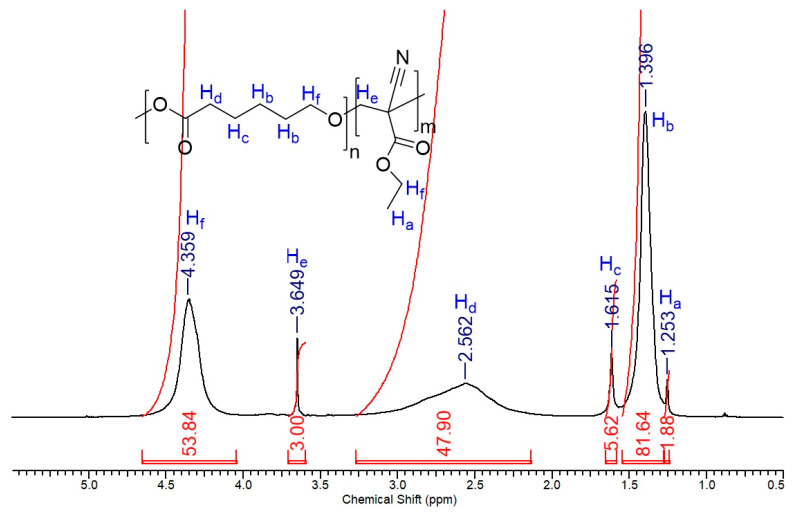
^1^H-NMR spectrum of the PECA-PCL copolymer (CDCl_3_, 600.17 MHz).

In the ^1^H-NMR spectrum of the PECA-PCL copolymer (Figure 3), the signal at *d* 1.25 ppm is assigned to H_a_ hydrogens in methyl groups. The broad signal at d 1.0–1.50 ppm corresponds to the H_b_ hydrogens as well as the signals for H_c_ (*d* 1.58–1.65 ppm), H_d_ (*d* 2.1–3.2 ppm), and H_f_ (d 4.35 ppm) in the long chain of methylene groups, respectively. The singlet at d 3.64 ppm is assigned to the H_e_ hydrogens. Finally, H_f_ methylene hydrogens present signals at d 4.2–4.4 ppm, characteristic of methylene attached to oxygen atoms in ester fragments. The absence of carboxy- and olefinic hydrogen signals belonging to PCL and ethyl 2-cyanoacrylate in the spectrum confirms those absent in the IR spectra shown above.

Also, the ^13^C{^1^H} NMR spectrum presents signals at *d* 13.29, 13.42, and 43.29 ppm, characteristic of the upfield methyl and methylene groups far in connectivity to oxygen atoms, but the signals at *d* 64.33 and 70.45 ppm are assigned to methylene carbons attached directly to oxygen atoms. The lonely and low intensity signal at *d* 29.66 ppm reflects a quaternary carbon, due to the absence of hydrogens attached to it. Signals at 114.75 ppm (C≡N carbon) and 165.51 ppm (carbonyl carbons) complete the assignment.

It is worth mentioning that intensities and integrated areas of upfield signals H_b_, H_c,_ and H_d_ are proportionally greater in area than signals of H_a_ and H_e_, which suggests a big ratio of polycaprolactone segment to ethyl 2-cyanoacrylate segment in the polymer structure.

The degree of polymerization (DP) [13,14], for repeating units *n* and *m* (RU1) and (RU2), respectively, was calculated as the ratio of DP of RU1 and RU2, illustrated in Figures S12–S14 and Tables S8 and S9 of the Supplementary Material, which yielded a value of 2383.80 g·mol^−1^.

#### 2.1.5. Thermal Analysis

**Figure 4:**
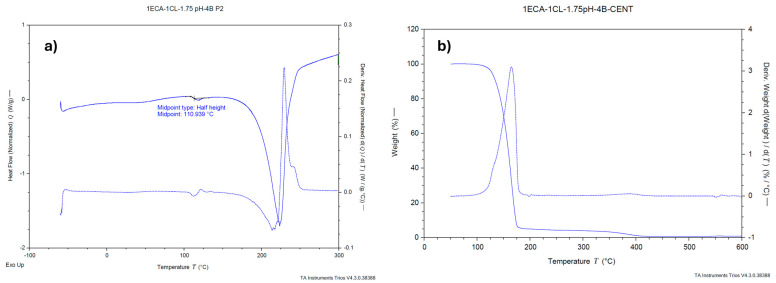
(**a**) Thermogram of sample 1PECA:1PCL, 4% Brij20, pH 1.75. (**b**) TGA of sample 1PECA:1PCL, 4% Brij20, pH 1.75.

In Figure 4a, the thermogram of the PECA-PCL copolymer obtained through DSC is shown in order to determine the glass transition temperature (*T*_g_). Based on the known properties of each monomer, copolymerization can be inferred. The monomer ethyl-2-cyanoacrylate has a *T*_g_ of 115 °C [15], while polycaprolactone has a *T*_g_ of −60 °C [16]. The *T*_g_ of the PECA-PCL was 110.9 °C, indicating that copolymerization occurred, with poly(ethyl cyanoacrylate) being predominant. Figure 4b shows the result of the thermogravimetric analysis, in which two significant weight losses can be observed. The first corresponds to a weight loss of approximately 90%, starting just above 100 °C and ending around 180 °C, attributed to the poly(ethyl cyanoacrylate). The second, smaller weight loss of around 5% begins at 180 °C and is attributed to the presence of both monomers in the sample.

#### 2.1.6. Particle Size Determined Using a Transmission Electron Microscope (TEM)

**Figure 5:**
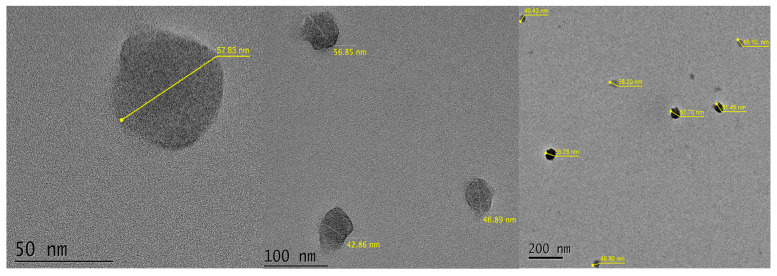
Micrographs of sample 1PECA:1PCL, 4% Brij20, pH 1.75.

In Figure 5, it can be observed that after sonicating the solution and placing a drop, the nanoparticles were sufficiently dispersed, resulting in an isolated nanoparticle with an average size of 51.74 nm. The micrograph images and measurement details are provided in Tables S10 and S11 of the Supplementary Material.

## 3. Materials and Methods

### 3.1. Synthesis of (PECA-PCL) Nanoparticles

Different proportions of PECA, PCL, and surfactants were evaluated to determine the optimal conditions for achieving the desired particle size. Specifically, four surfactants were employed: polysorbate 80 (Tween 80) (Sigma-Aldrich, St. Louis, MO, USA, Batch AO112i, CAS 9005-65-6), Alkonat L70 (Oxiteno, San Juan del Rio, QRO, Mexico, Batch OCZ2017009907, CAS 3055-97-8), Genapol LRO (Clariant, Ecatepec, EDO.MEX, Mexico, Batch MXAC029202), and Brij-20 (Sigma-Aldrich, St. Louis, MO, USA, Batch MKCM1739, CAS 9005-00-9). Table 2 shows the different proportions used. After selecting the surfactant, the effect of pH values (1.5, 1.75, and 2.0) on microemulsion formation, particle size, and uniformity was assessed [17]. These pH values were chosen because a higher proton concentration in strongly acidic media promotes more effective electrostatic repulsion between forming particles due to the stabilization of the electric double layer [18,19,20]. This phenomenon contributes to the formation of smaller nanoparticles with narrower size distributions. A one-way analysis of variance (ANOVA) was performed to determine significant differences among the surfactants used and to evaluate the effect of pH variation within the established range.

#### 3.1.1. Ring-Opening of ε-Caprolactone

**Figure 6:**
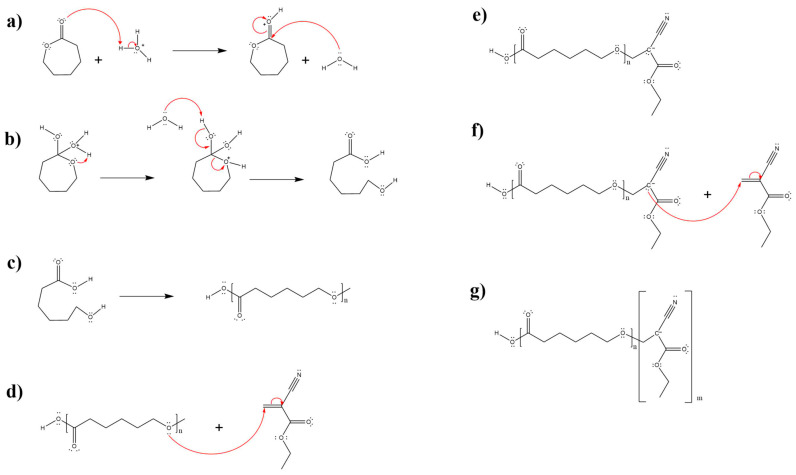
Ring-opening of ε-caprolactone and the copolymerization mechanism (PECA-PCL): (**a**) Protonation of the carbonyl oxygen atom, (**b**) ring-opening of ε-caprolactone through nucleophilic attack, (**c**) polycaprolactone (PCL), (**d**) nucleophilic attack on the alkene carbon, (**e**) bond formation between both monomers and generation of a carbanion, (**f**) nucleophilic attack of the carbanion on an electrophilic carbon, and (**g**) bond formation between both monomers and generation of a new carbanion.

The ring-opening of the ε-caprolactone monomer was carried out via acid-catalyzed hydrolysis of the cyclic ester (lactone) [21]. Initially, the ester was activated toward nucleophilic attack through protonation of the carbonyl oxygen atom [22], followed by nucleophilic addition of water (Figure 6a). This resulted in the formation of a tetrahedral intermediate, which subsequently underwent ester bond cleavage. The proton transfer step converted the OR’ group into an effective leaving group (Figure 6b). As a result of this mechanism, polycaprolactone (PCL) was obtained as the main product (Figure 6c), which was later used in the copolymerization with ethyl-2-cyanoacrylate.

#### 3.1.2. Synthesis of (PECA-PCL) Nanoparticles

Distilled water was acidified with HCl until the desired pH was reached [13]. The specified percentage of the monomer ε-caprolactone (Sigma-Aldrich, St. Louis, MO, USA, Batch MKBX5175V CAS 502-44-3), as shown in Table 2, was then added, and the mixture was stirred for 25 min to promote the ring-opening of the ε-caprolactone structure [23], as explained in Section 3.1.1. After the stirring time was completed, the specified percentage of the selected surfactant (see Table 2) was weighed and added. Stirring was continued until the solution became homogeneous. The solution was transferred to a Schlenk flask properly prepared for a vacuum-nitrogen system, and an inert atmosphere was established with N_2_(g) throughout the entire polymerization process. The first dose of the monomer ethyl-2-cyanoacrylate (Sigma-Aldrich, St. Louis, MO, USA, Batch SLBK1428V, CAS 7085-85-0) was added in a semicontinuous manner every 30 min over a period of 3 h (6 doses) [24].

#### 3.1.3. Copolymerization Mechanism (PECA-PCL)

The copolymerization mechanism of PCL and PECA begins with a nucleophilic attack on the alkene moiety of the ethyl cyanoacrylate molecule (Figure 6d). As a result of this attack, the electrons from the double bond migrate along the carbon chain, generating a carbanion and forming a bond between the two monomers (Figure 6e). The presence of the carbanion enables a subsequent nucleophilic attack on another molecule of ethyl cyanoacrylate (Figure 6f), leading to the formation of an additional bond between the monomers and the growth of the copolymer chain (Figure 6g).

### 3.2. Characterization of Nanoparticles

#### 3.2.1. Micelle Size Measurement

Micelle size was measured by quasi-elastic light scattering (QLS) using a Zetasizer Nano ZS90 system (Malvern Panalytical, Malvern, UK). This instrument uses a 633 nm He–Ne laser and operates at a detection angle of 90°, suitable for particle size measurements in the range of 0.3 nm to 5 µm. A 1 mL aliquot of each emulsion sample was placed in a glass cuvette, and measurements were performed using the QLS system under standard operating conditions.

#### 3.2.2. Micelle Breakdown and Surfactant Removal

After completing the microemulsion, a 50/50 *v*/*v* ethanol/copolymer solution was prepared [25]. The solution was centrifuged using a Thermo Scientific ST 8 device (Thermo Scientific, Waltham, MA, USA) at 9000 rpm for 30 min. Upon completion, the supernatant was removed, and the precipitate (nanoparticles) was retained. The precipitate was washed three times with ethanol [25], with centrifugation at 9000 rpm for 15 min between each wash and agitation of the solution. Once the final precipitate was obtained, it was dried at 40 °C for 2 h. After drying, the nanoparticles were ground using a mortar and pestle to break up agglomerates before proceeding to characterization. At the end of this stage, white powder-form nanoparticles were obtained. To verify the effectiveness of the ethanol washes and centrifugation in removing the surfactant (and thus confirming the breakdown of the microemulsion), the synthesized copolymer, the pure surfactant, and the washed copolymer were analyzed using a Thermo Scientific Nicolet iS50 FT-IR spectrophotometer (Thermo Scientific, Waltham, MA, USA) and ^1^H and ^13^C{^1^H}NMR spectroscopy. Spectra were recorded on a JEOL JNM-ECA600 600 MHz spectrometer (JEOL Ltd., Akishima, Tokyo, Japan), operating at 600.17 for ^1^H and 150.91 MHz for ^13^C (proton decoupled).

#### 3.2.3. Thermal Analysis Procedure

The dry nanoparticles were analyzed using a thermogravimetric analyzer and the Discovery differential scanning calorimeter model from TA-Instruments (Waters LLC, New Castle, DE, USA). For the analysis of the glass transition temperature (*T*_g_), the sample was placed in a hermetic aluminum capsule with a heating rate of 5 °C·min^−1^, within a range of −100 °C to 300 °C in the differential scanning calorimeter. For the thermogravimetric analysis, approximately 10 mg of the sample was placed in a platinum cell, and temperature increments of 5 °C·min^−1^ were performed within a range of 0 °C to 600 °C. Both experiments were carried out under a nitrogen flow of 50 cm^3^·min^−1^.

#### 3.2.4. Particle Size Analysis Using a Transmission Electron Microscope (TEM)

A transmission electron microscope (TEM), JEM-JEOL 2100 (JEOL Ltd., Akishima, Tokyo, Japan), operated at 200 kV, was used for nanoparticle imaging. For sample preparation, a small amount of nanoparticles was dispersed in 10 mL of 2-propanol and sonicated for 10 min to ensure uniform dispersion. A drop of the resulting suspension was deposited onto a carbon-coated copper grid and allowed to dry at room temperature prior to analysis.

## 4. Conclusions

There is a significant difference in micelle size when using the surfactants Tween80, Alkonat L70, Genapol LRO, and Brij20. Brij20 allows for the synthesis of PECA-PCL copolymeric nanoparticles with micelle sizes smaller than 100 nm. The ideal surfactant concentration to achieve micelle sizes under 100 nm is 4%, despite no significant difference being found between the 3%, 4%, and 5% concentrations. A difference in micelle size was observed when varying the pH within the range of 1.5 to 2, with higher pH values resulting in smaller micelle sizes. However, a pH of 1.75 was selected because the micelle size distribution at pH 2 showed a wide variation in particle size. Thermal analysis using differential scanning calorimetry (DSC) and thermogravimetric analysis (TGA) confirmed the occurrence of copolymerization. This was evidenced by a shift in the glass transition temperature (*T*_g_) of the copolymer (110 °C) in the DSC thermogram, and by the mass loss in the TGA analysis, which indicates the presence of both polymers in the nanoparticles. The average size of the nanoparticles obtained using this methodology was 51.74 nm, confirmed through transmission electron microscope (TEM) images.

## Figures and Tables

**Table 1:** Summary of results for pH and surfactant concentration variation.

PECA-PCL	% Brij20	pH	Average Micelle Size ^a^
1:1	3	1.5	61.14
1:1	4	1.5	60.42
1:1	5	1.5	57.82
1:1	3	1.75	55.96
1:1	4	1.75	54.66
1:1	5	1.75	48.72
1:1	3	2	39.94
1:1	4	2	42.66
1:1	5	2	39.96

^a^ All measurements were performed in triplicate; therefore, the average values are reported.

**Table 2:** Proportions used of PCL, PECA, and surfactants (polysorbate 80 (Tween 80), Alkonat L70, Genapol LRO, and Brij-20) and micelle sizes obtained through QLS.

[PCL] [m/m]	[PECA] [m/m]	% Tween80	nm	Mean ^a^
2	1	5	502.2	
2	1	5	549	530.5
2	1	5	540.2	
1	1	5	96.84	
1	1	5	96.76	96.7
1	1	5	96.49	
2.5	0.5	5	737.4	
2.5	0.5	5	801	754.1
2.5	0.5	5	723.8	
1	1	6	203.1	
1	1	6	209.5	205.5
1	1	6	203.8	
2	1	7	179.5	
2	1	7	176.4	179.0
2	1	7	181	
1	1	7	274.2	
1	1	7	278.1	276.3
1	1	7	276.6	
		% Alkonat L70		
2	1	5	279.4	
2	1	5	302.5	294.0
2	1	5	300	
		% Genapol LRO		
2	1	5	739.8	
2	1	5	1135	928.8
2	1	5	911.6	
1	1	5	1451	
1	1	5	1653	1823.3
1	1	5	2366	
		% Brij-20		
2	1	5	74.08	
2	1	5	75.04	74.0
2	1	5	72.76	
1	1	5	52.85	
1	1	5	52.85	51.9
1	1	5	50.13	

^a^ Each measurement was performed in triplicate; therefore, the reported micelle size is shown as an average.

## Data Availability

The original contributions presented in this study are included in the article/Supplementary Materials. Further inquiries can be directed to the corresponding author(s).

## Supplementary Materials

The following supporting information can be downloaded at https://www.mdpi.com/article/10.3390/molecules30132668/s1. Table S1. ANOVA for Micelle Size by Surfactant; Table S2. Means for Micelle Size by Surfactant with 95.0% Confidence Intervals; Table S3. Multiple Range Tests for Micelle Size by Surfactant—Method: 95.0 Percent LSD; Table S4. Analysis of Variance for Micelle Size—Type III Sum of Squares; Table S5. Least Squares Means for Micelle Size with 95.0% Confidence Intervals; Table S6. Multiple Range Tests for Micelle Size by Surfactant %—Method: 95.0 Percent LSD; Table S7. Multiple Range Tests for Micelle Size by pH—Method: 95.0 Percent LSD; Table S8. 1H and 13C NMR correlation for PECA-PCL copolymer; Table S9. Calculations for DP ratio in RU1 and RU2; Table S10. TEM micrographs of nanoparticles of sample 1PECA:1PCL, 4% Brij20, pH 1.75; Table S11. Nanoparticle size determined by TEM for sample 1PECA:1PCL, 4% Brij20, pH 1.75; Figure S1. Mean plot for micelle size vs. surfactant percentage; Figure S2. Mean plot for micelle size vs. pH; Figure S3. Micelle size distribution plot for synthesis at pH 1.5 and 3% surfactant; Figure S4. Micelle size distribution plot for synthesis at pH 1.75 and 3% surfactant; Figure S5. Micelle size distribution plot for synthesis at pH 2 and 3% surfactant; Figure S6. Micelle size distribution plot for synthesis at pH 1.5 and 4% surfactant; Figure S7. Micelle size distribution plot for synthesis at pH 1.75 and 4% surfactant; Figure S8. Micelle size distribution plot for synthesis at pH 2 and 4% surfactant; Figure S9. Micelle size distribution plot for synthesis at pH 1.5 and 5% surfactant; Figure S10. Micelle size distribution plot for synthesis at pH 1.75 and 5% surfactant; Figure S11. Micelle size distribution plot for synthesis at pH 2 and 5% surfactant; Figure S12. 1H-NMR spectrum of PECA-PCL copolymer (CDCl3, 600.17 MHz); Figure S13. 13C-NMR spectrum of PECA-PCL copolymer (CDCl3, 150.91 MHz); Figure S14. Repeating units for PECA-PCL copolymer.

## Abbreviations

The following abbreviations are used in this manuscript:

PECA	poly(ethyl cyanoacrylate)
PCL	poly(ε-caprolactone)
