# Supplementary material for: Semicontinuous Microemulsion Polymerization of Polymeric Nanoparticles of Poly(cyanoacrylates) and Poly(caprolactone)
Source: Molecules. 2025 Jun 20;30(13):2668. doi: 10.3390/molecules30132668 (PMC12251225; doi:10.3390/molecules30132668)
Supplement: Supplementary file 1 [file molecules-30-02668-s001.zip › molecules-3680882-supplementary.pdf]

Supplementary Material

Semicontinuous Microemulsion Polymerization of Polymeric Nanoparticles of Poly(cyanoacrylates) and Poly(caprolactone)

Gerardo León-Sánchez,[1] Eulogio Orozco-Guareño,[2a] Oscar Guillermo Zúñiga-González,[2b] Luisa Fernanda Briones-Márquez,[2c], Raúl R. Quiñonez-López[2d], J. Baudelio Campos-García,[3] María de Jesús Palacios-Sánchez [2,*]

[1]*Departamento de Procesos Tecnológicos e Industriales, Instituto Tecnológico de Estudios Superiores de Occidente, Periférico Sur Manuel Gómez Morín #8585 C.P. 45604, Guadalajara, Jalisco, México, gleon@iteso.mx*
[2]*Laboratorio de Fisicoquímica, Departamento de Química, Centro Universitario de Ciencias Exactas e Ingenierías, Universidad de Guadalajara, Blvd. Marcelino García Barragán # 1421, esq. Calzada Olímpica, 44430, Guadalajara, Jalisco, México.* [a]eulogio.orozco@academicos.udg.mx, [b]guillermo.zuniga@academicos.udg.mx, [c]fernanda.briones@academicos.udg.mx
[3]*Departamento de Química, Universidad Autónoma de Aguascalientes, Av. Universidad # 940, Ciudad Universitaria,C.P. 20100, Aguascalientes, Aguascalientes, México, jesus.campos@edu.uaa.mx*

**maria.palacios1333@academicos.udg.mx*

The Supplementary Material includes the statistical analysis of one-way ANOVA for Micelle Size by Surfactant, Multiple Range Tests for Micelle Size by pH, scatter plots showing different surfactant percentages and pH variation, IR and NMR analyses, and TEM micrographs of the nanoparticles.

Table S1. ANOVA for Micelle Size by Surfactant

Source	Sum of Squares	df	Mean Square	F-Ratio	P-Value
Between Groups	1.4355E6	3	478499.	48.54	**0.0000**
Within Groups	78859.4	8	9857.42		
Total (Corrected)	1.51436E6	11			

Table S2. Means for Micelle Size by Surfactant with 95.0% Confidence Intervals

			Std. Error		
**Surfactant**	**Cases**	**Mean**	**s Pooled**	**Lower Limit**	**Upper Limit**
Tween 80	3	96.6967	57.322	3.22776	190.166
Alkonat L70	3	293.967	57.322	200.498	387.436
Genapol LRO	3	928.8	57.322	835.331	1022.27

Brij20	3	73.96	57.322	19.5089	167.429
Total	12	348.356			

Table S3. Multiple Range Tests for Micelle Size by Surfactant – Method: 95.0 Percent LSD

Surfactant	Cases	Mean	Homogeneous Groups
Brij20	3	73.96	X
Tween80	3	96.6967	X
Alkonat L70	3	293.967	X
Genapol LRO	3	928.8	X

The Multiple Range Test shows whether there is a significant difference between each of the variables; in this case, it indicates whether there is a significant difference between the surfactants used. The results are presented based on the "X" or homogeneous groups.

Table S4. Analysis of Variance for Micelle Size – Type III Sum of Squares

Source	Sum of Squares	df	Mean Square	F-Ratio	P-Value
MAIN EFFECTS					
A:pH	553.616	2	276.808	77.30	**0.0006**
B: % Surfactant	26.559	2	13.2795	3.71	0.1227
RESIDUAL	14.3231	4	3.58078		
TOTAL (CORRECTED)	594.498	8			

Table S5. Least Squares Means for Micelle Size with 95.0% Confidence Intervals

			Error	Limit	Limit
**Level**	**Cases**	**Mean**	**Std.**	**Lower**	**Upper**
OVERAL MEAN	9	51.2489			
pH					
1.5	3	59.79	1.09252	56.7567	62.8233
1.75	3	53.1067	1.09252	50.0733	56.14
2	3	40.85	1.09252	37.8167	43.8833
%Surfactant					
3	3	52.3433	1.09252	49.31	55.3767
4	3	52.58	1.09252	49.5467	55.6133
5	3	48.8233	1.09252	45.79	51.8567

Table S6. Multiple Range Tests for Micelle Size by Surfactant % – Method: 95.0 Percent LSD

%Surfactant	Cases	LS Mean	LS Sigma	Homogeneous Groups
5	3	48.8233	1.09252	**X**
3	3	52.3433	1.09252	**X**
4	3	52.58	1.09252	**X**

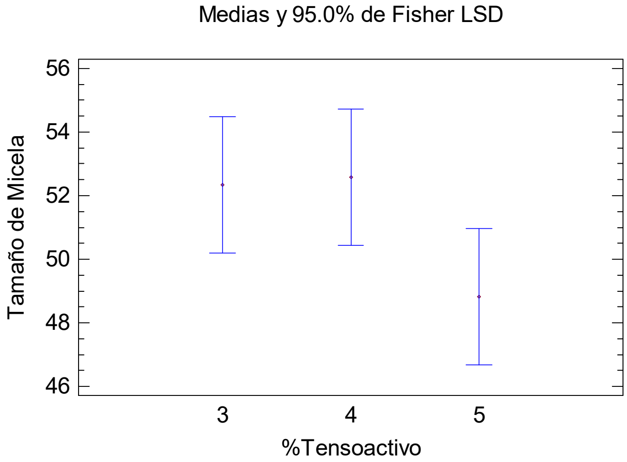

Figure S1. Mean plot for micelle size vs. surfactant percentage.

Table S7. Multiple Range Tests for Micelle Size by pH – Method: 95.0 Percent LSD

pH	Casess	LS Mean	LS Sigma	Homogeneous Groups
2	3	40.85	1.09252	X
1.75	3	53.1067	1.09252	X
1.5	3	59.79	1.09252	X

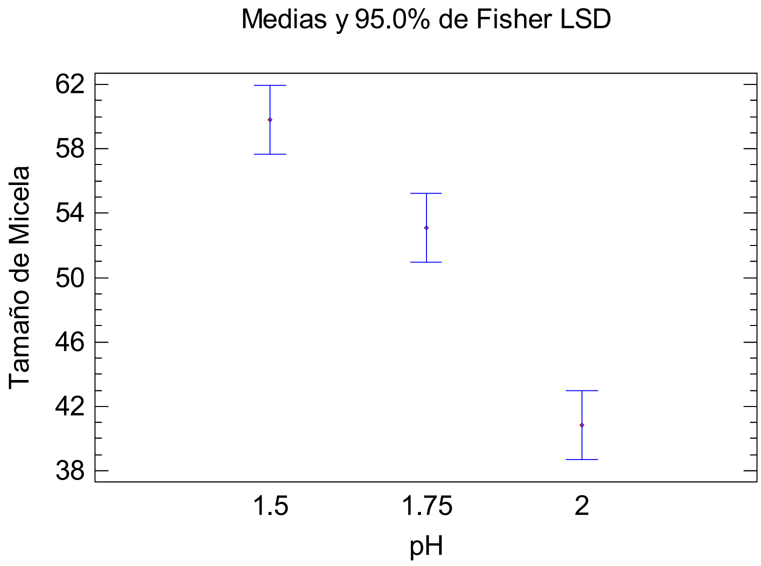

Figure S2. Mean plot for micelle size vs. pH.

Scatter plots of different surfactant percentages and pH variation

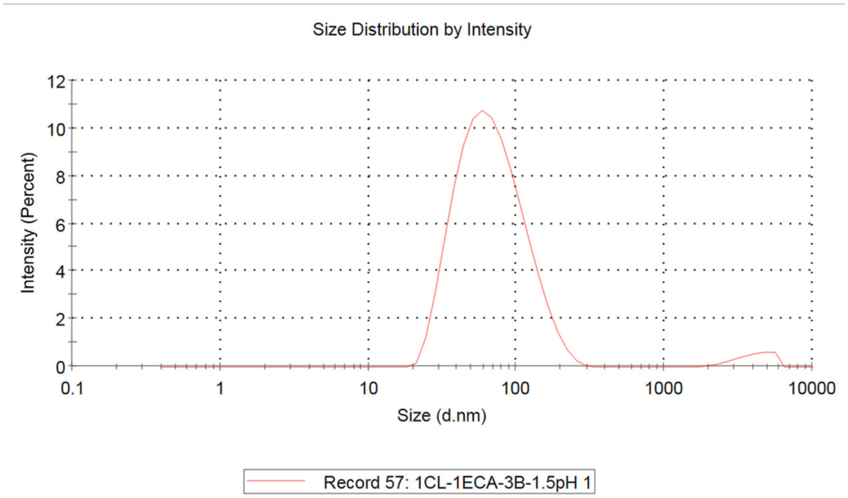

Figure S3. Micelle size distribution plot for synthesis at pH 1.5 and 3% surfactant.

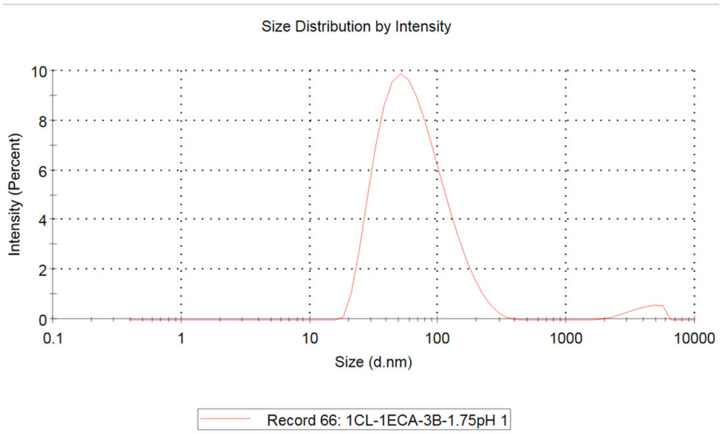

Figure S4. Micelle size distribution plot for synthesis at pH 1.75 and 3% surfactant.

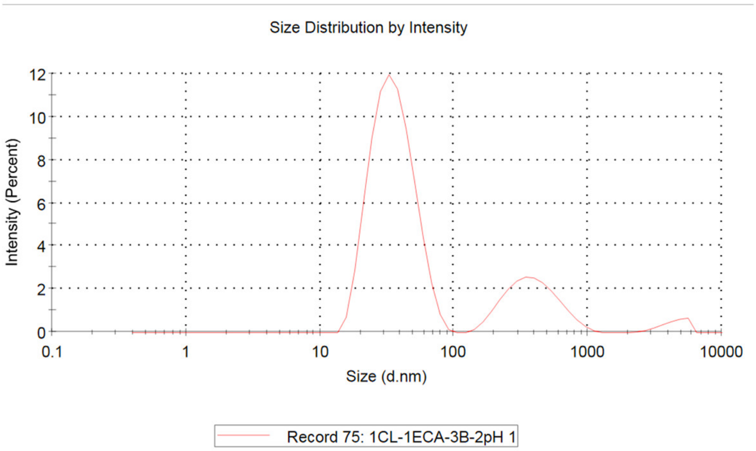

Figure S5. Micelle size distribution plot for synthesis at pH 2 and 3% surfactant.

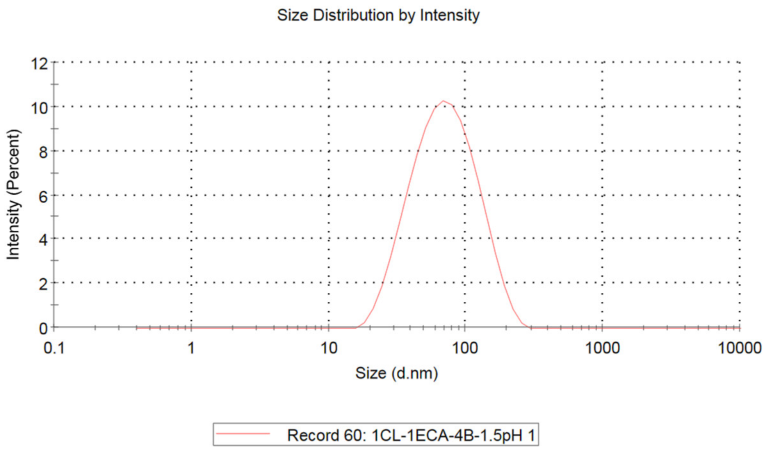

Figure S6. Micelle size distribution plot for synthesis at pH 1.5 and 4% surfactant.

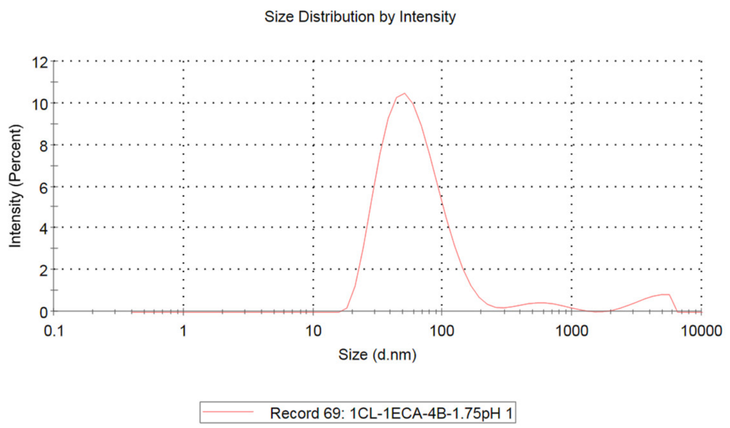

Figure S7. Micelle size distribution plot for synthesis at pH 1.75 and 4% surfactant.

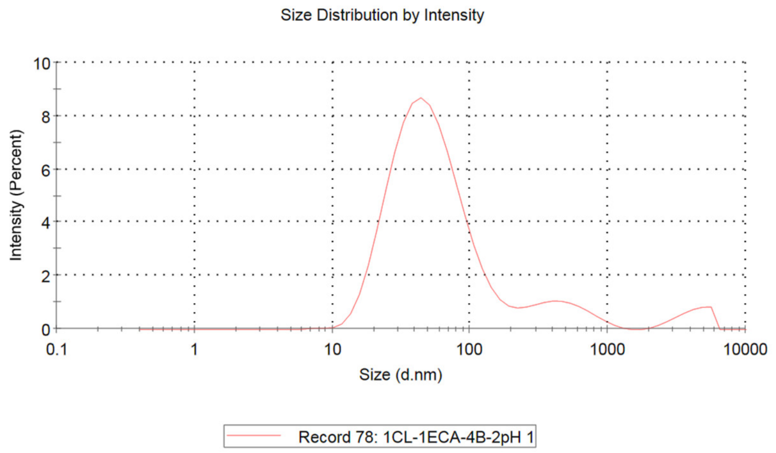

Figure S8. Micelle size distribution plot for synthesis at pH 2 and 4% surfactant.

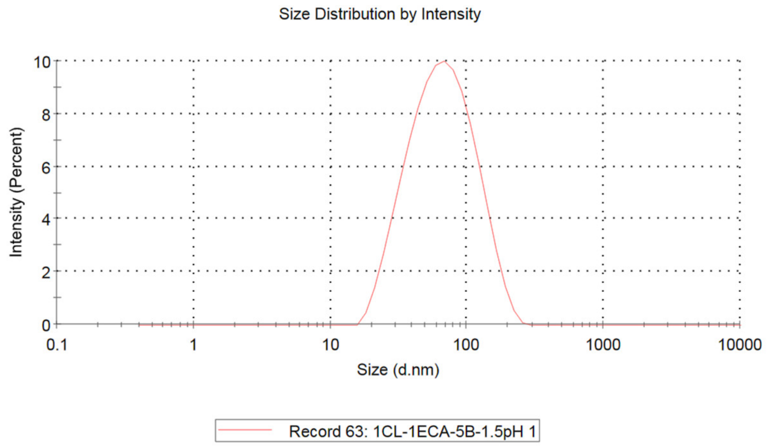

Figure S9. Micelle size distribution plot for synthesis at pH 1.5 and 5% surfactant.

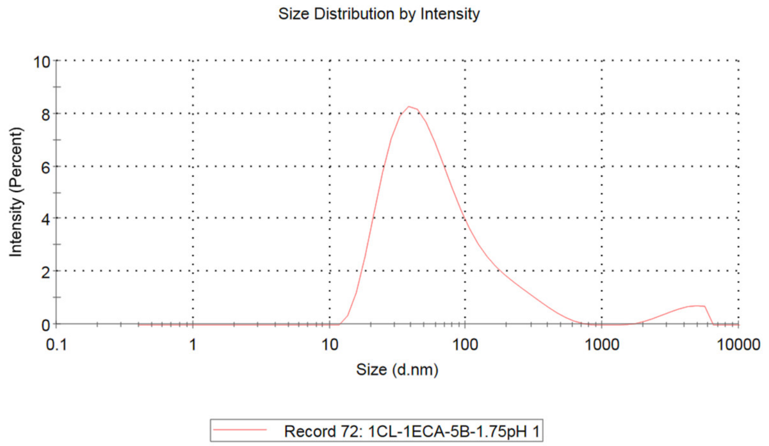

Figure S10. Micelle size distribution plot for synthesis at pH 1.75 and 5% surfactant.

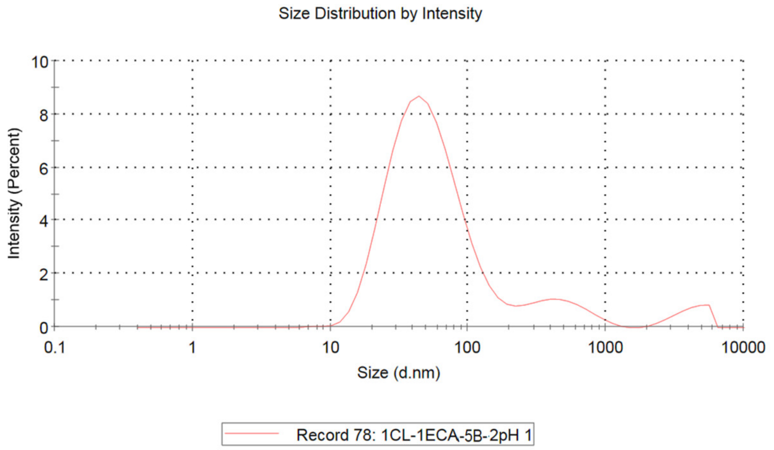

Figure S11. Micelle size distribution plot for synthesis at pH 2 and 5% surfactant.

^1H and $^{13}C\{^1H\}$NMR spectra were recorded in a JEOL JNM-ECA600 600 MHz spectrometer operating at 600.17 (for ^1H) and 150.91 MHz (for ^{13}C, proton decoupled). Chemical shifts are reported in parts per million relative to $SiMe_4$, using the residual solvent signals: $CDCl_3$ (δ_H 7.26 ppm, δ_C 77.0 ppm). A total of 16 transients were recorded for each spectrum at 17.7 °C.

NMR spectra were acquired from a solution of 100 mg of copolymer dissolved in 700 µL of $CDCl_3$ at 25 °C.

^1H and ^{13}C-NMR spectroscopy characterization

Table S8. ^1H and ^{13}C NMR correlation for PECA-PCL copolymer.

position	δ (ppm) ^1H RMN	δ (ppm) ^1H RMN (peak apex)	δ (ppm) ^{13}C RMN
H-a (H_3C-, unit m)	1.25	1.25	A, 13.82
H-b (-CH_2-, unit n)	1.30 - 1.50	1.39	B 13,29
H-c (-CH_2-, unit n)	1.58 - 1.65	1.61	C 13.42
H-d (-CH_2-C=O, unit n)	2.1 – 3.2	2.56	D 43.29
H-e (-CH_2-, unit n)	3.6 – 3.7	3.64	E, 70.45
H-f (-CH_2-, unit m)	4.1 – 4.60	4.35	F 64.33
---	---	---	G 29.66
---	---	---	C≡N 114.75
---	---	---	C=O 165.51

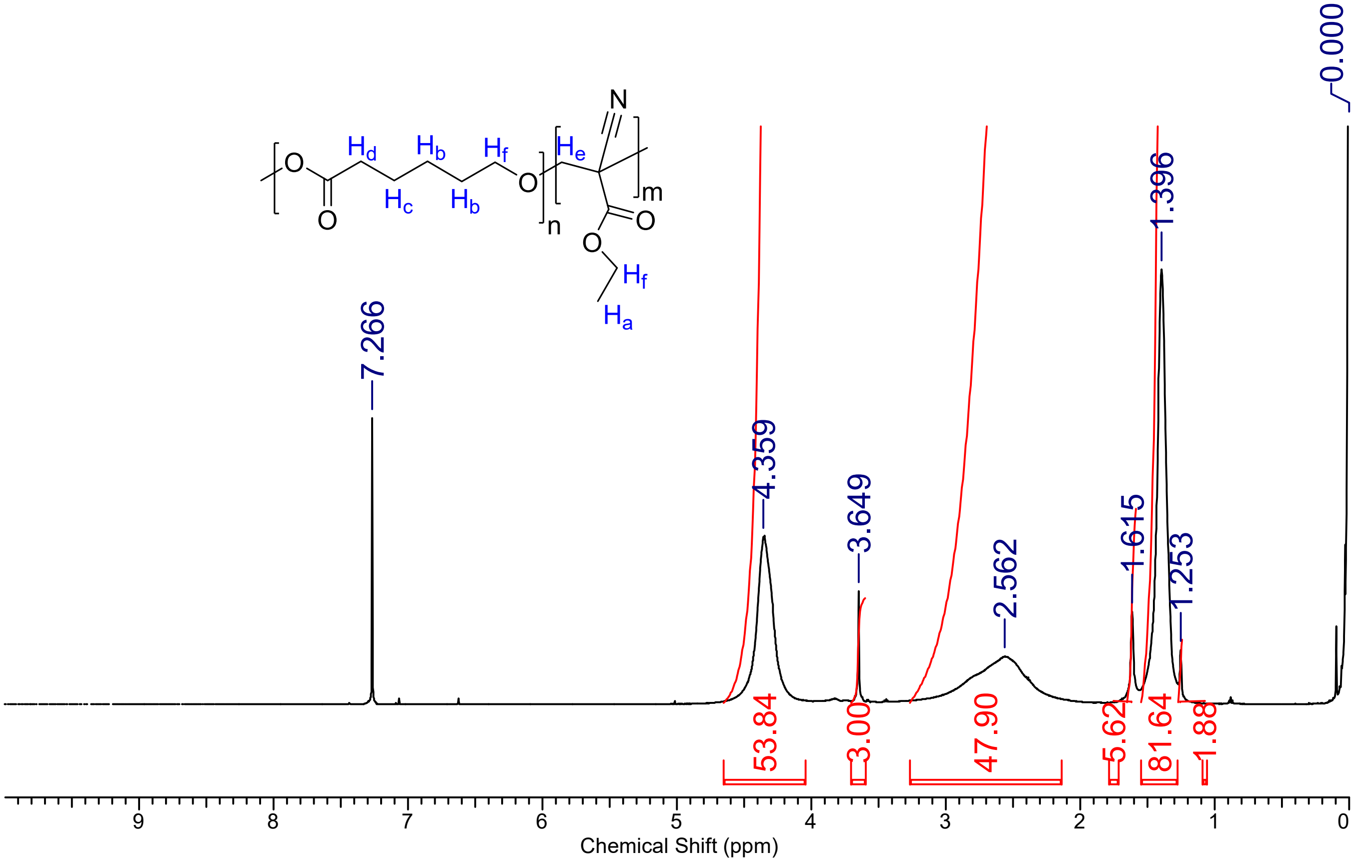

Fig. S12. ^1H-NMR spectrum of PECA-PCL copolymer ($CDCl_3$, 600.17 MHz).

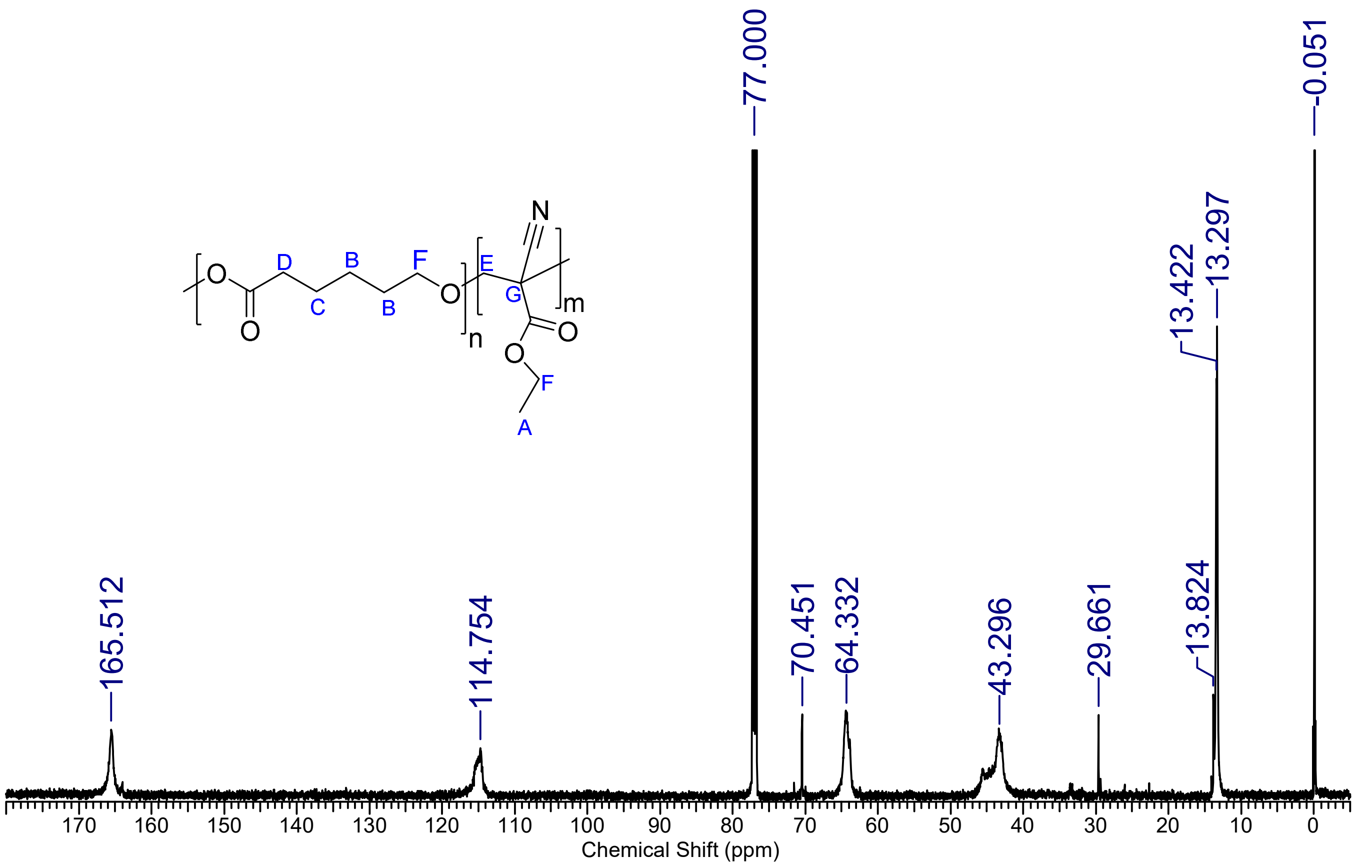

Fig. S13. ^{13}C-NMR spectrum of PECA-PCL copolymer ($CDCl_3$, 150.91 MHz).

Molecular Weight determination for PECA-PCL copolymer

The degree of polymerization ratio (DP_1/DP_2) for each repeating unit, was calculated and summarized in table S-2, according to the ratio of selected hydrogens in repeating unit 1 (RU1) to selected hydrogens in repeating unit 2 (RU2) illustrated in Figure S-3 with the formula:

$$\frac{DP_{RU1}}{DP_{RU2}} = \frac{\frac{\text{Sum of integral areas of the repeating unit 1}}{\text{number of H of the repeating unit 1}}}{\frac{\text{Sum of integral areas of the repeating unit 2}}{\text{number of H of the repeating unit 2}}}$$

Hydrogens corresponded to the H_f methylene groups in the structure shown in Figure S-1 were not selected due the signal at δ 4.1-4.60 ppm (peak apex at 4.35 ppm) includes all of the H_f hydrogens in these methylene groups (2 protons in repeating unit *n* (RU1) and 2 protons in repeating unit *m* (RU2)). For this reason, its corresponding integral area was not selected in calculations.

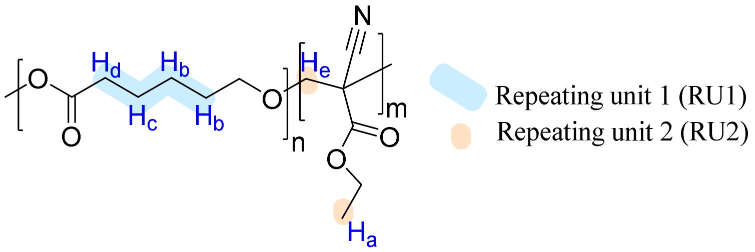

Fig. S14. Repeating units for PECA-PCL copolymer.

Table S9. Calculations for DP ratio in RU1 and RU2.

Repeating unit	Color in structure	Sum of integral areas	Number of hydrogens	DP	DP ratio $\frac{DP_{RU1}}{DP_{RU2}}$
RU1	Blue	135.16[a]	8	16.90	17.23
RU2	Orange	4.88[b]	5	0.98	
[a]Calculated as: 81.64+5.62+47.90. [b]Calculated as: 3.00+1.88					

Thus, the estimated molecular weight of the polymer structure containing 17.23 RU1 plus one RU2 is calculated as:

$$MW(polymer\ structure) = 17.23 * \ MW(RU1) + 1.00 * MW\ (RU2)$$

Where: MW is the molecular weight for RU1, RU2 and the polymer structure. Calculation yields:

$$= 17.23 * 130.16\ g * mol^{-1} + 1.00 * 141.14\ g * mol^{-1}$$

$$=2383.80\ g * mol^{-1}$$

References

Daria Kaczmarek, Jakob S.Diget, Bo Nyström, Gergő Gyulai, Róbert Mészáros, Tibor Gilányi, Imre Varga, *Colloids and Surfaces A:Physicochemical and Engineering Aspects*, *532* (**2017**), 290–296. https://doi.org/10.1016/j.colsurfa.2017.04.078.

Table S10. TEM micrographs of nanoparticles of sample 1PECA:1PCL, 4% Brij20, pH 1.75

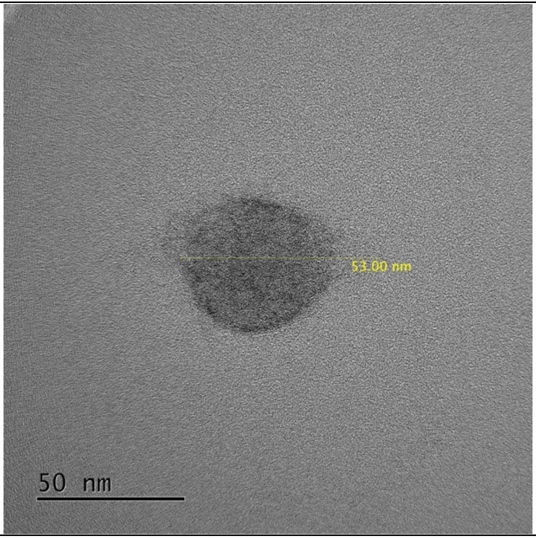

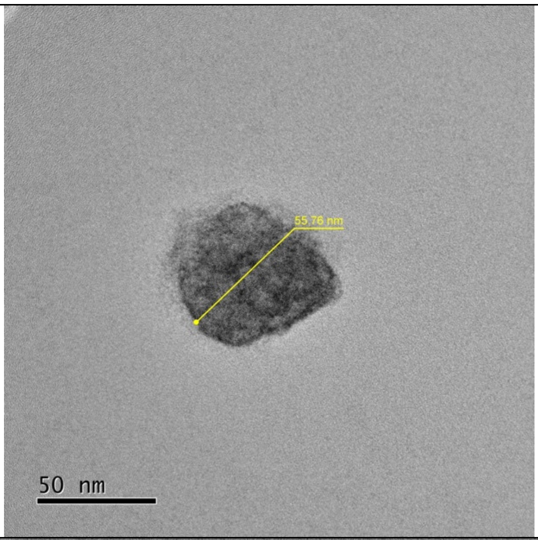

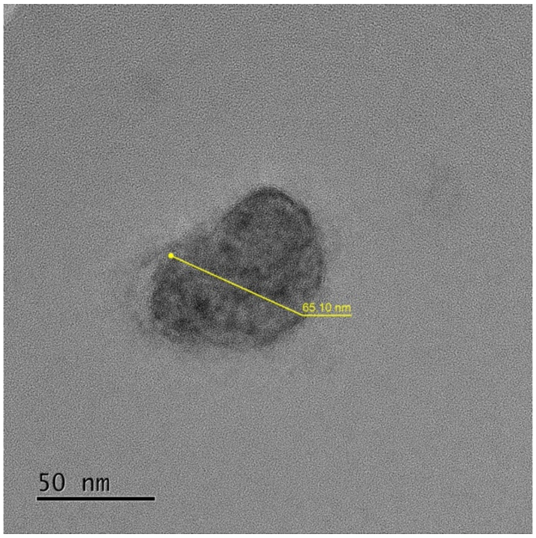

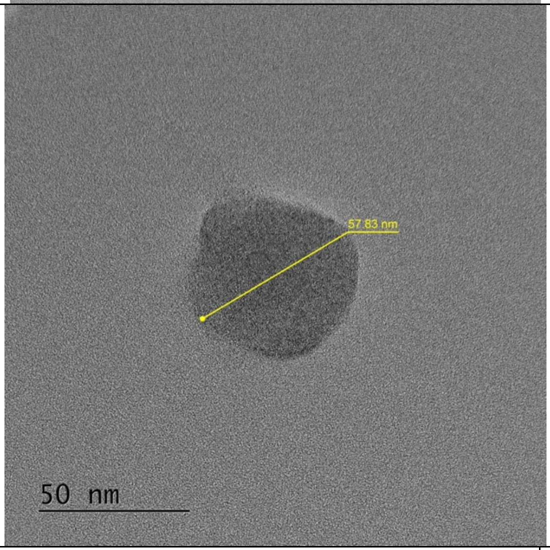

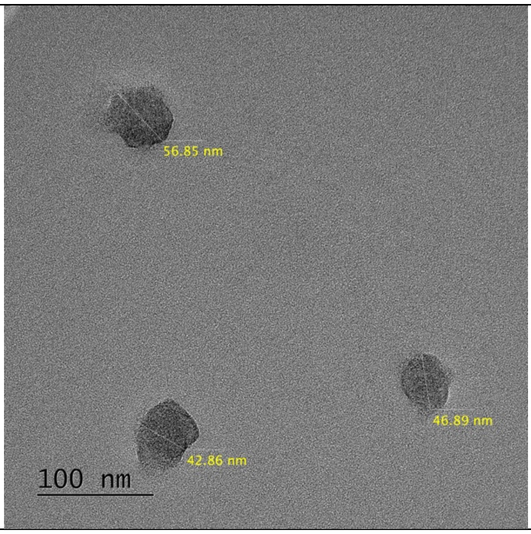

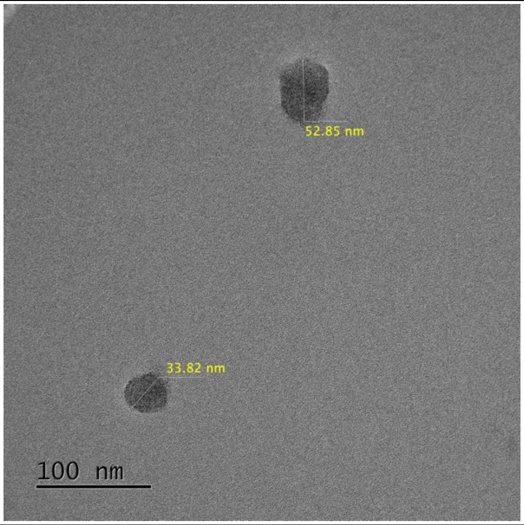

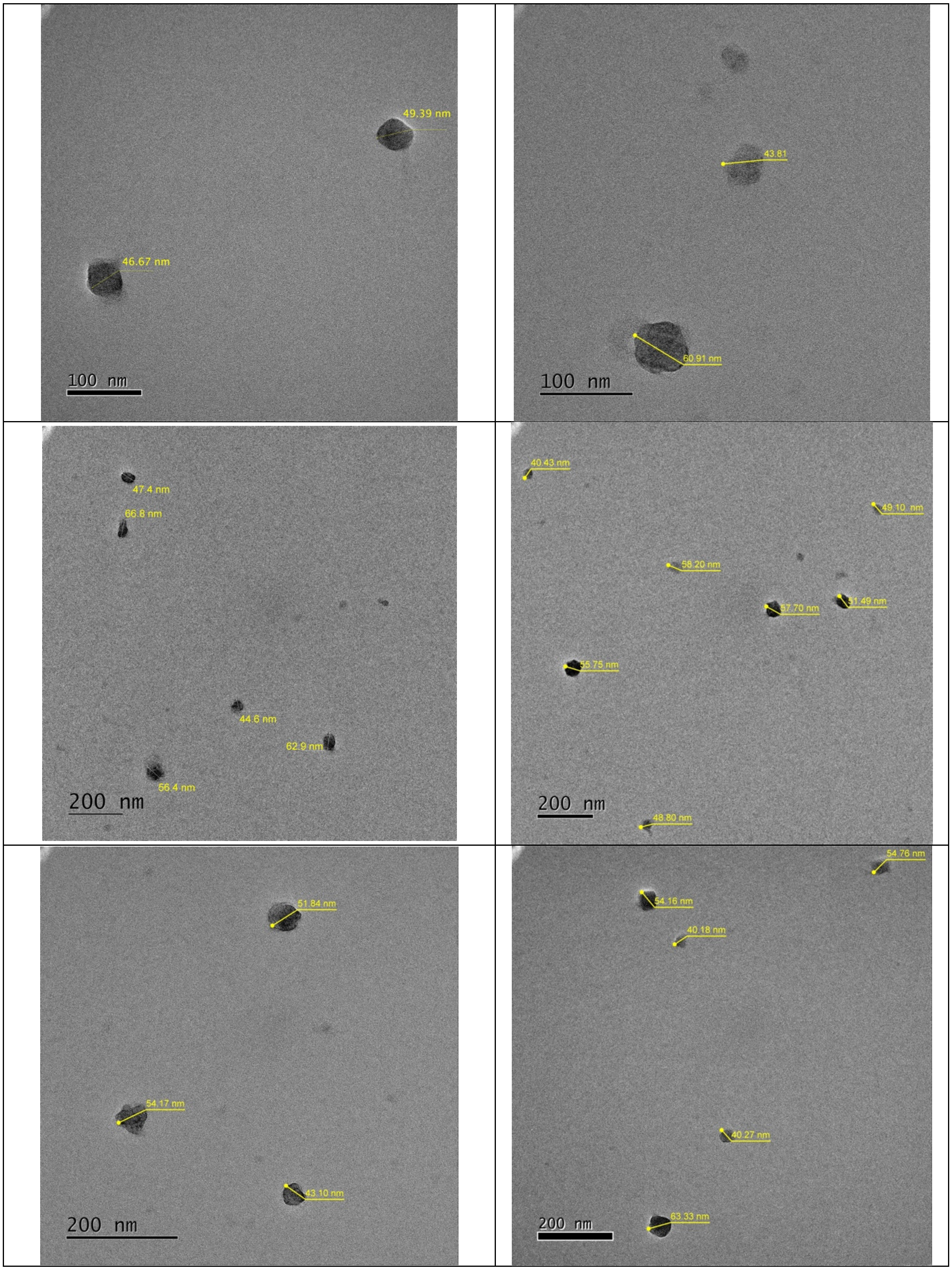
49.39 nm
46.67 nm
100 nm
43.81
60.91 nm
100 nm
47.4 nm
66.8 nm
44.6 nm
62.9 nm
56.4 nm
200 nm
40.43 nm
49.10 nm
58.20 nm
57.70 nm
51.49 nm
55.75 nm
48.80 nm
200 nm
51.84 nm
54.17 nm
43.10 nm
200 nm
54.76 nm
54.16 nm
40.18 nm
40.27 nm
63.33 nm
200 nm

Table S11. Nanoparticle size determined by TEM for sample 1PECA:1PCL, 4% Brij20, pH 1.75

Micrograph	Nanoparticle	Size nm
1	1	53.00
2	1	55.76
3	1	65.09
4	1	57.83
5	1	56.85
	2	42.86
	3	46.90
6	1	33.82
	2	52.85
7	1	46.67
	2	49.39
8	1	60.91
	2	43.82
9	1	47.40
	2	66.89
	3	56.44
	4	44.67
	5	62.99
10	1	55.75
	2	57.70
	3	51.49
	4	48.80
	5	58.21
	6	40.43
	7	49.11
11	1	51.84
	2	54.17
	3	43.09
12	1	63.33
	2	40.27
	3	40.18
	4	54.16
	5	54.76
	Mean	51.74
